# Effective photocatalysis and toxicity investigations in zebra fish embryos using highly selective hydrogen sensors and supercapacitor electrode materials for SnO_2_–PANI nanocomposites

**DOI:** 10.1039/d5na00783f

**Published:** 2026-01-19

**Authors:** Amutha Eswaran, Rajaduraipandian Subramaniam, Madhumitha Thirumalainambi, Gurusamy Annadurai, Vijayalakshmi Shankar

**Affiliations:** a SPKCEES, Manonmaniam Sundaranar University Alwarkurichi – 627 412 India annananoteam@gmail.com; b Sri Paramakalyani College, Manonmaniam Sundaranar University Alwarkurichi – 627412 India; c School of Healthcare Science and Engineering, Vellore Institute of Technology Vellore – 14 India vijimicro21@gmail.com

## Abstract

The current study presents the synthesis of binary hybrid tin oxide–polyaniline (SnO_2_–PANI) nanocomposites, which have potential applications in gas sensing, energy storage, photocatalysis, and biomedicine. Ternary hybrid formation is confirmed by a number of characterization studies, including X-ray diffraction (XRD), thermogravimetric analysis (TGA), particle size analysis (PSA), Fourier transform infrared (FTIR) spectroscopy, scanning electron microscopy with energy dispersive X-ray (SEM–EDAX) analysis, and atomic force microscopy (AFM). The nanoparticles showed good gas sensing capability, with a sensitivity for hydrogen (H_2_) at 50 ppm. The electrode material exhibited a sensing capacity of 94% and a stability period of 50 days. The electrochemical performance of the binary hybrid is revealed by a three-electrode setup using a potassium hydroxide (KOH) (3 M) electrolyte. The particles exhibited a large surface area and a high current density, according to the electrochemical study. The bimetallic nanoparticles are used to degrade the rhodamine dye in a photocatalytic chamber. The nanoparticles removed 92% of the dye within 180 minutes. The model animal showed only mild toxicity to the nanoparticles in the toxicity test, which was conducted to ascertain whether the photocatalyst is hazardous. The particles showed good activity against both Gram-positive and Gram-negative bacteria compared to the control in the antibacterial test.

## Abstract 2

Binary hybrid tin oxide–polyaniline (SnO_2_–PANI) nanocomposites, which have potential uses in gas sensing, energy storage, photocatalysis, and biomedicine, are synthesized in the present work (created with https://www.biorender.com.

## Graphical abstract

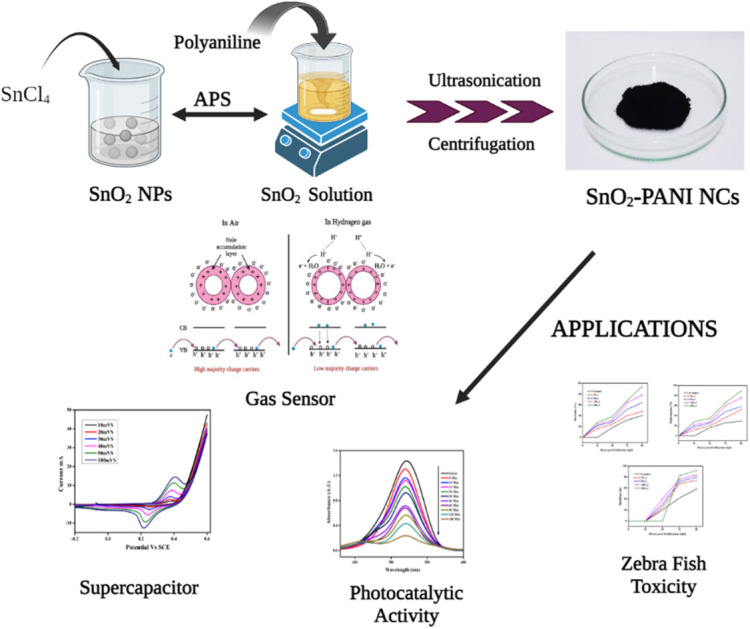

## 1. Introduction

In recent times, inorganic–organic nanocomposites have become a very interesting field of study because of their distinct physical and chemical properties.^1^ Tin oxide is a well-known substance that is utilized in gas sensors and dye-based solar cells due to its high band gap and n-type semiconductor nature.^2^ Polyaniline has garnered attention because of its superior electrical conductivity, environmental stability, and ease of synthesis. Two of the advantages of SnO_2_ are its cost-effectiveness and eco-friendliness. It is crucial to develop and study composite materials that combine the benefits of SnO_2_ and polyaniline (PANI) for electrochemical applications. Although SnO_2_ exhibits promising sensing and electrochemical properties, its poor conductivity significantly restricts its performance. Conducting polymers, such as polyaniline (PANI), offer an effective strategy to mitigate these drawbacks by providing rapid charge transport pathways and flexible surface chemistries. Therefore, integrating SnO_2_ with PANI can yield a synergistic composite with enhanced functional characteristics.^3,4^

In particular, the conducting polymers have reduced the operating temperature to approximately room temperature, which has enhanced the gas detection potential.^5^ The conducting polymer polyaniline (PANI) has been studied as a potential material for gas sensing applications (or electroactivity) because of its intriguing redox chemistry, controllable electrical conductivity, and environmental stability.^6^ PANI has garnered significant attention due to its notable features for H_2_ gas detection at low operating temperatures, making it a useful conducting polymer.^7^

The transition metal oxide hydrous ruthenium dioxide has been found to be one of the most promising electrode materials for electrochemical capacitors because of its various advantages, including its capacity to reversibly store charges through redox reactions.^8,9^ In lithium-ion batteries, dye-sensitized solar cells, transparency conducting electrodes, electrochromic windows, flat-panel displays, catalytic supports, catalyst transistors, rechargeable batteries, and other applications, nanoscale SnO_2_ has shown promise as a gas sensor and electrode.^5,10,11^ It is commonly known that SnO_2_ exhibits effective semiconductivity in addition to conventional redox properties, setting it apart from other conventional supports used in catalytic applications.^12^

In contrast, PANI–tin oxide nanocomposites have enhanced conductivity and thermal stability, making them ideal for flexible electronics and energy storage.^13^ PANI–metal oxide composites, such as those with TiO_2_ or ZnO, exhibit boosted photocatalytic activity and environmental stability, making them useful in sensors and anticorrosion coatings. PANI–tin oxide nanocomposites have improved mechanical and barrier properties and are suitable for coatings and packaging.^14^

It is still challenging for the SnO_2_ photocatalyst's photocatalytic efficiency to meet the demands of practical use because of electron–hole pair recombination.^15^ Improving its photocatalytic performance is necessary to meet the demands of commercial applications.^16^ The photocatalytic performance of the SnO_2_ nanoparticles can be improved by changing the SnO_2_ surface to generate smaller particles and a lower bandgap energy.^17^

In this work, SnO_2_–PANI nanocomposites were successfully synthesized through an *in situ* polymerization approach. Comprehensive physicochemical analyses, including XRD, FTIR spectroscopy, SEM–EDX, AFM, TGA, and PSA, confirm the crystalline structure, chemical interactions, elemental composition, surface morphology, thermal stability, and particle size distribution of the materials. The electrochemical performance of the synthesized nanocomposites was evaluated using cyclic voltammetry (CV), galvanostatic charge–discharge (GCD), and electrochemical impedance spectroscopy (EIS), demonstrating their suitability as high-performance electrode materials. Additionally, the nanocomposites exhibited notable antimicrobial activity against selected bacterial strains, indicating their potential for multifunctional applications. Overall, the synergistic combination of SnO_2_ and PANI enhances both electrochemical behavior and antimicrobial efficacy, suggesting that these nanocomposites may be promising candidates for advanced sensor technology and bio-protective applications.

## 2. Materials and methods

Ammonium persulphate ((NH_4_)_2_S_2_O_8_), anhydrous sodium carbonate (Na_2_CO_3_), tin chloride (SnCl_2_), sodium hydroxide (NaOH), aniline (C_6_H_5_NH_2_), ≥ 99.5% pure hydrochloric acid (HCL), nitric acid (HNO_3_), sulfuric acid (H_2_SO_4_), and ethanol (C_2_H_5_OH) were purchased from Sigma-Aldrich. Every reagent that was bought was of analytical grade, and it was used without any additional purification. Aqueous solutions were prepared for each experiment using distilled water. The MTCC, Chandigarh, provided us with bacterial isolates of Gram-negative *Pseudomonas fluorescens* and *Enterobacter* and Gram-positive *Staphylococcus aureus*.

### 2.1. Synthesis of tin oxide (SnO_2_) nanoparticles

The method involved creating distinct solutions of hydrous stannous chloride and anhydrous sodium carbonate in distilled water. The stannous chloride solution and hydrous sodium carbonate solution were prepared in a 1 : 2 M ratio. The stannous chloride solution was added to a 1000 mL beaker and continuously stirred at room temperature using a magnetic stirrer. A white precipitate was produced after five minutes of continuous stirring and drop-by-drop addition of the sodium carbonate solution. After stirring for another thirty minutes, the mixture was allowed to settle for four hours. The solution was first separated from the particles that had gathered at the bottom of the beaker by decantation, and then, it was filtered. To get rid of the impurities, ethyl alcohol was used for a final wash of the precipitate. To prepare SnO_2_ nanoparticles, the obtained product was dried for 12 hours at 60 °C and then calcined for 1 hour at 350 °C in a muffle furnace.^18^

(i) Precipitation of tin hydroxide from SnCl_4_ and NaOH:SnCl_4_ + 4NaOH → Sn(OH)_4_↓ + 4NaCl

(ii) Thermal dehydration/calcination of the hydroxide to SnO_2_:Sn(OH)_4_ → SnO_2_ + 2H_2_O

(iii) Combining the two steps:SnCl_4_ + 4NaOH → SnO_2_ + 4NaCl + 2H_2_O

(iv) SnCl_2_ oxidizes to SnO_2_, an illustrative overall equation (with O_2_ as the oxidant):SnCl_2_ + 1/2O_2_ → SnO_2_ + 2Cl^−^

### 2.2. Synthesis of polyaniline (PANI)

Aniline was oxidized with ammonium persulfate to form polyaniline. During this synthesis, a 0.5 M aniline solution in 0.5 M HCl was produced. Furthermore, 0.5 M ammonium persulphate was dissolved in distilled water to make a solution. A magnetic stirrer was used to agitate the aniline hydrochloride solution in a 500 mL beaker for approximately ten minutes. Subsequently, 0.5 M ammonium persulphate was progressively added to the solution while stirring continuously. Aniline hydrochloride's white solution progressively turned green. After stirring for another thirty minutes, the mixture was allowed to rest for two hours before being filtered. The result was a precipitate that was pale green and was referred to as polyaniline. We rinsed the precipitate several times with distilled water to get rid of contaminants and unreacted aniline.^18^ In the end, the precipitate was cleaned with acetone to get rid of the unreacted components. The finished product was dried for 12 hours at 50 °C. Using a mortar and pestle, the dried material was ground into a fine powder.

(i) Protonation:C_6_H_5_NH_2_ + HCl → C_6_H_5_NH_3_^+^ + Cl^−^

(ii) Oxidation:C_6_H_5_NH_3_^+^ + S_2_O_8_^2−^ → [C_6_H_5_NH_2_]˙^+^ + 2SO_4_^2−^ (generation of an aniline radical cation)

(iii) Coupling of radical cations → oligomers → chain growth → polyaniline (emeraldine salt).

### 2.3. Synthesis of the SnO_2_–PANI nanocomposites

A 500 mL beaker was filled with a dissolved aniline solution in hydrochloric acid, and it was agitated for five minutes. After that, 0.5 g (10 wt%) of SnO_2_ nanoparticles was added, and a magnetic stirrer was used to agitate the mixture for around 15 minutes. After stirring for an additional ten minutes and adding ammonium persulphate drop by drop, the precipitate was left to settle for 30–40 minutes. To get rid of the contaminants, the precipitate was filtered and then repeatedly cleaned with purified water. Following acetone cleaning and a 24 hour drying process at 50 °C, the precipitate was pulverized.

### 2.4. Electrochemical measurement

The working electrode (WE), made of a Ni foam, had an exposed geometric area of 1 cm^2^. The Ni foam was cut into pieces of 1 cm × 1 cm, thoroughly cleaned, and used as a substrate for electrode fabrication. The electrochemical behavior of the SnO_2_–PANI nanocomposites was examined using cyclic voltammetry (CV), galvanostatic charge–discharge (CD) measurements, and electrochemical impedance spectroscopy (EIS). The working electrode was made by combining the nanocomposite materials, conductive carbon (carbon black) and the binder polytetrafluoroethylene (PTFE) in a weight ratio of 85 : 15 : 10. The resultant electrode paste (∼2 mg) was applied to a Ni foam current collector that had already been prepared in order to use it as a working electrode. Electrochemical studies were performed in a 3 M potassium hydroxide electrolyte using a three-electrode cell, where Ag/AgCl was the reference electrode and a Pt foil was the counter electrode. Different scan and current rates were employed in CV and CD investigations. The discharge profile was utilized to compute the specific capacitance, energy density, and power density of the system through the application of the subsequent formulae:1
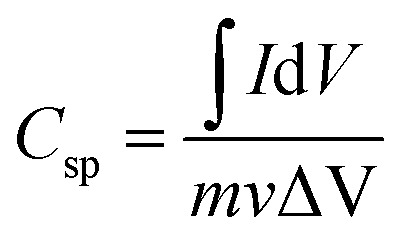
2
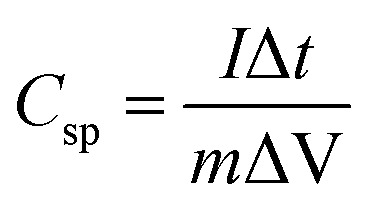
where “*M*” is the mass of the active material, “*S*” is the scan rate, “∫*I*d*V*” is the absolute surface area, and “Δ*V*” is *V*_2_ − *V*_1_, *i.e.*, the potential window.

The energy density (*E*) and power density (*P*) were calculated based on the electrochemical data obtained from galvanostatic charge–discharge (GCD) measurements. Specifically, (i) the energy density (*E*) was calculated using the discharge time (Δ*t*) obtained from the GCD curve and the specific capacitance (C) determined using the three-electrode setup, as expressed in eqn (3):

3 *E* = 1/2 *CV*^2^,

where *V* is the operating potential window, and (ii) the power density (*P*) was subsequently calculated using the discharge time and energy density values:

4 *P* = *E*/Δ*t*

### 2.5. Fabrication of the gas-sensing electrode

The active substrate for the fabrication of gas-sensing electrodes was printed circuit boards (PCBs). The PCB's conducting layer had to be cleaned before a 1 cm^2^ area on it could be taken and drawn with a positive resist to prepare the contact channel. Next, it was dipped in a FeCl_3_ solution to complete the etching procedure. To remove the resist substance, acetone was utilized. Using a spin coater set to 4000 rpm for 5 minutes, the substrate was finally covered with the sensing material (SnO_2_–PANI nanocomposites). The electrodes were dried for thirty minutes at 80 °C in a vacuum oven. In order to connect the single-core wires to the voltage source for sensor measurements, they were linked at the terminals.

### 2.6. Antibacterial property

#### 2.6.1. Agar well diffusion assay

Gram-positive *Staphylococcus aureus* and Gram-negative *Enterobacter* and *Pseudomonas fluorescens* were used as pathogenic bacteria in the well diffusion method to examine the SnO_2_–PANI nanocomposites' antibacterial activity. The antibacterial activity of the bacterial species indicated above was assessed using different concentrations of 25, 50, 75, and 100 µL. Following a 24-hour treatment at 37 °C for each plate, the zone of bacterial inhibition was identified.

### 2.7. Photocatalytic activity

The photocatalytic performance of the SnO_2_–PANI nanocomposites was evaluated by measuring the photocatalytic degradation of rhodamine dye under UV irradiation. The standard procedure involved adding 0.1 g SnO_2_–PANI nanocomposite was added to 100 mL of an aqueous rhodamine dye solution with a starting concentration of 1 ppm. To achieve adsorption/desorption equilibrium, the SnO_2_–PANI nanocomposite suspension and dye solution were shaken for thirty minutes in the dark before exposure to radiation. Then, the suspension was subjected to UV light. At regular intervals of thirty minutes, about two milliliters of the suspension were taken out of the mixture and centrifuged to separate the photocatalyst particles during irradiation. A UV-vis spectrophotometer was used to quantify the concentration of the rhodamine solution in the supernatant. This instrument had a unique absorbance at *λ*_max_ = 550 nm.

The following formula was used to determine the degradation efficiency: 
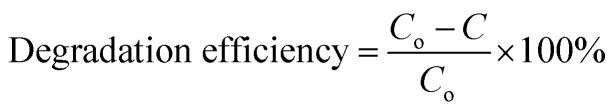
where *C*_o_ is the starting concentration of the rhodamine dye and *C* is the concentration of the rhodamine dye solution after degradation time “*t*”.

### 2.8. Fish maintenance and the SnO_2_–PANI nanocomposite exposure

Wild zebrafish (*Danio rerio*) were collected from the Fisheries Department in Madurai, India. For a month, the fish were acclimated in the zebrafish tank at SPKCEES, Manonmaniam Sundaranar University in Alwarkurichi, India. The glass aquarium had a 50 L capacity and was kept at a constant temperature of about 28 °C with a light–dark cycle of 14:10 hours (6.8–8.5). Fish were fed live *Artemia nauplii* from Inve Aquaculture Nutrition, Thailand, once a day and commercial dry flake food called Basic Flake from China twice a day. A single female zebrafish and two male zebrafish in a single breeding tank were mated to produce embryos. To facilitate reproduction, males and females were physically separated from one another at the same time of the day by a clear block. This barrier was then removed the next morning. To get rid of any remaining material from their surface, the eggs were rinsed several times with the E3 medium. The fertilized eggs in the 6, 12, 24, and 48-well culture plates were immediately supplemented with 20 embryos in 2 milliliters of solution per well. Each experimental treatment and control group was duplicated three times. The SnO_2_–PANI nanocomposites at various concentrations (0, 25, 50, 75, 100, 250 µg mL^−1^) were cultivated in healthy fertilized embryos for 24–96 hours post fertilization. Every 24 hours, groups exposed to the SnO_2_–PANI nanocomposites had their dead embryos removed from the plates. Each experimental plate was covered with a foil and maintained at 28 °C to keep light from penetrating.

### 2.9. Embryo toxicity test

Using a stereomicroscope, the zebrafish embryo's embryonic developmental stage was examined during the whole exposure time after fertilization. The embryos were exposed to different doses of the SnO_2_–PANI nanocomposites (0, 25, 50, 100, and 200 µg mL^−1^) for a period of 24–96 hpf. The rate of hatching and the rate of embryonic death were measured every 24 hours. The term “hatching rate” refers to the proportion of viable embryos left in each well after hatching. Photographs and documentation of the deformities in the embryos and larvae from the treatment and control groups were taken. The malformed embryos were imaged using a stereomicroscope, and the percentage of malformed embryos was recorded every 24 hours.

## 3. Results and discussion

### 3.1. UV-vis absorption spectroscopy and bandgap measurement

**Fig. 1:**
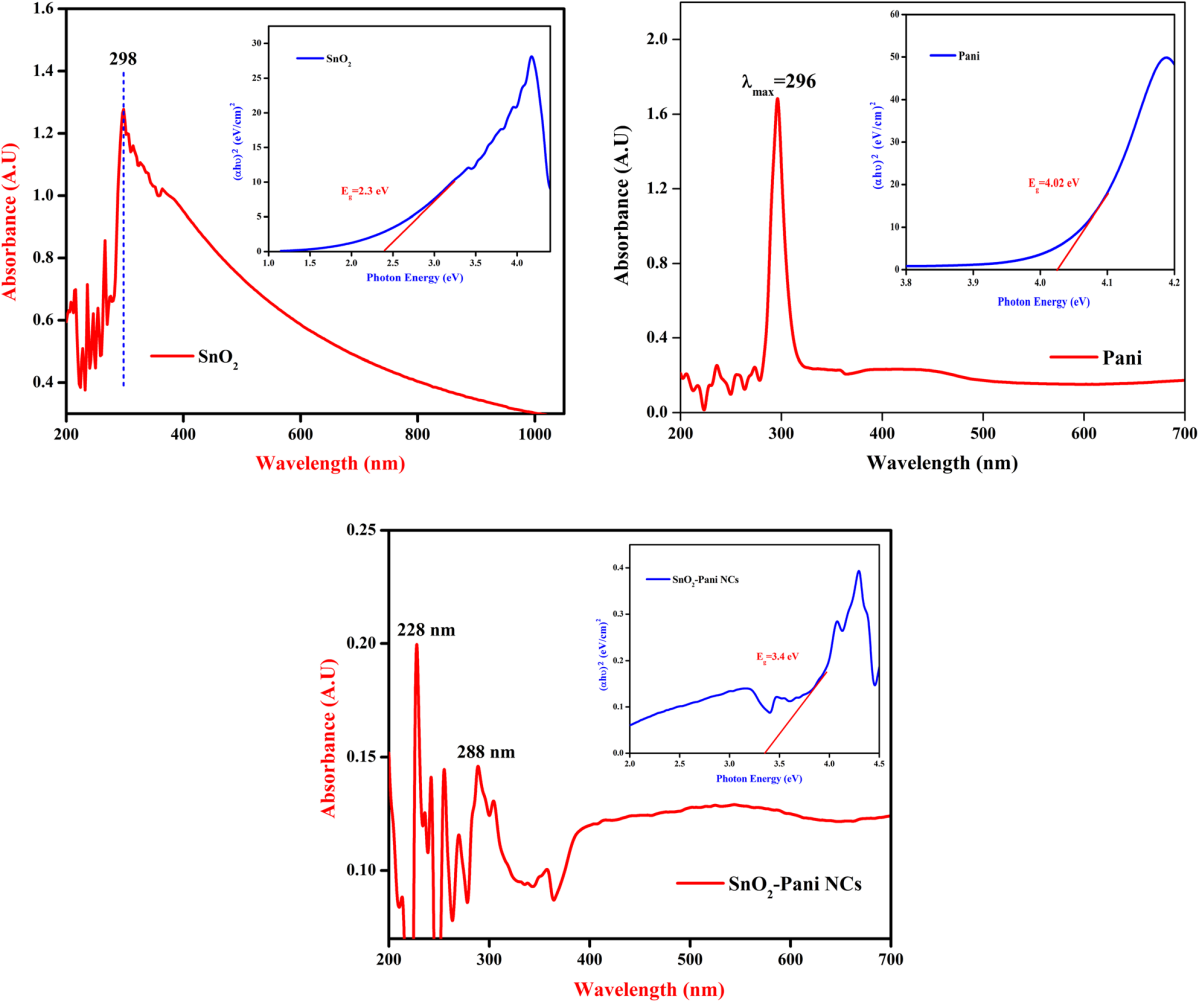
UV-vis spectra and Tauc plots of tin oxide, polyaniline and the SnO_2_–PANI nanocomposites.

The UV-vis study was carried out to determine the optical bandgap and to understand the optical behavior of the synthesized materials. The UV-vis absorbance spectra of SnO_2_–PANI, polyaniline, and tin oxide nanocomposite materials are displayed in Fig. 1. The synthesized materials exhibit a clear absorption of visible and ultraviolet light. The tin oxide's light absorbance peak, located at 298 nm in the UV range, is attributed to the π → π* transition of C–C bonds, while the polyaniline peak, located at 296 nm, signifies the n → π* transition, as shown in Fig. 1. The Tauc plot inset in each graph was used to calculate the bandgaps of 2.3 eV (SnO_2_) and 4.02 eV (PANI).^19^ The SnO_2_–PANI nanocomposites exhibit UV absorption signals with a band edge of 288 nm and a high absorption intensity of 228 nm, with a corresponding bandgap of 3.4 eV. The red shifts in the peaks show substantial coupling reactions in the composite particle signals relative to their individual spectra, which are likewise related to the quantum confinement or quantum size effects.^20^ Additionally, the materials' bandgap is significantly changed when polyaniline is incorporated.^21^

The optical bandgap was calculated using the Tauc relation:(*αhν*)^*n*^ = *A* (*hν* − *E*_g_)where *α* is the absorption coefficient, *hν* is the photon energy, *A* is a constant, *E*_g_ is the optical bandgap, and *n* depends on the nature of the electronic transition (for a direct allowed transition, *n* = 2).

### 3.2. Fourier transform infrared spectroscopy

**Fig. 2:**
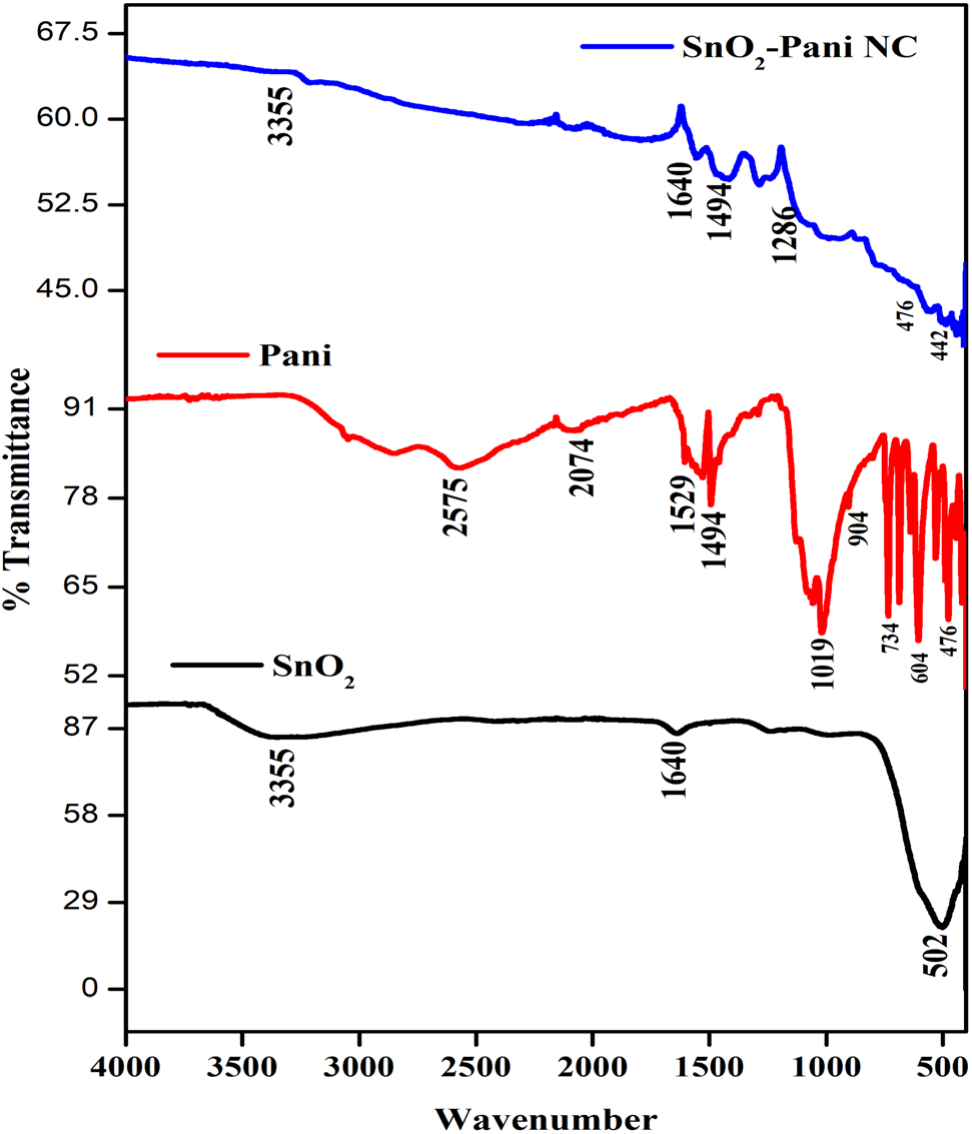
FT-IR spectra of tin oxide, polyaniline and the SnO_2_–PANI nanocomposites.

Fig. 2 shows the synthesized materials' FTIR spectra. We determined which functional groups interacted with the metal oxide atoms in polyaniline. The OH, C=C, and C–X groups are primarily responsible for the peaks seen in the FTIR spectrum of tin oxide, which are located at 3355 cm^−1^, 1640 cm^−1^, and 502 cm^−1^, respectively. For PANI–SnO_2_ nanocomposites, the OH group's stretching mode is responsible for the strong absorption peak at 3355 cm^−1^. The C=C, C–H, C–N, and C–Br stretching modes are responsible for the peaks at 1640 cm^−1^, 1494 cm^−1^, 1286 cm^−1^, 476 cm^−1^, and 442 cm^−1^, respectively.^22^ The interatomic vibrations produced the stretching band of polyaniline, which is detected at 2575 cm^−1^.

The peaks at 1529 cm^−1^, 1494 cm^−1^, 1019 cm^−1^, 904 cm^−1^, 734 cm^−1^, 604 cm^−1^, and 476 cm^−1^ correspond to –C=C–, C–H, C–N, C–C, C–Br, C–H, and C–X, respectively. When compared to the FTIR spectra of tin oxide, polyaniline, and SnO_2_–PANI nanocomposites, all of the samples had higher transmittance values. This indicates that the bonding properties of the PANI–SnO_2_ nanocomposites are affected by the reduction of the nanocomposites and the incorporation of metal oxide.^23^ The presence of dopants in the mixed nanocomposite results in peak shifting both downward and upward, which is related to the binding of the functional groups of the SnO_2_–PANI nanocomposites with the metal oxides.^24^

### 3.3. Structural analysis

**Fig. 3:**
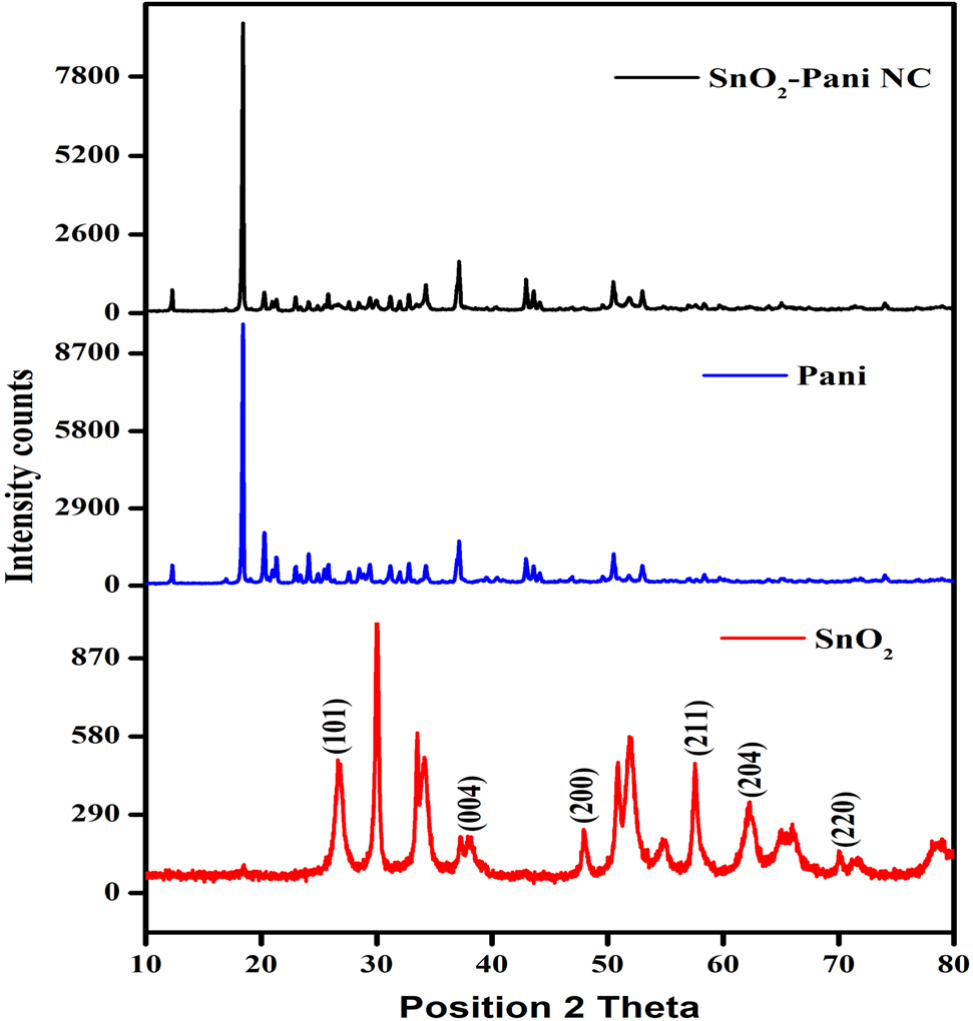
XRD patterns of tin oxide, polyaniline and the SnO_2_–PANI nanocomposites.

Through powder X-ray diffraction, phase purity and crystallographic data were obtained for SnO_2_ and SnO_2_ modified by polyaniline nanocomposites. The XRD patterns of PANI, SnO_2_–PANI, and tin oxide nanocomposites are displayed in Fig. 3.

Diffraction peaks are visible for (101), (004), (200), (211), (204), and (220) in the XRD pattern of tin oxide nanoparticles. Four broad peaks can be seen in the XRD pattern of polyaniline at 2*θ* values of 18°, 20°, 35°, and 50°, suggesting that the substance is amorphous. When the observed XRD peaks for the two samples are compared, it is evident that the peaks for (101), (004), (200), (211), and (220), with a primitive tetragonal structure, are matched with the observed nanocrystalline SnO_2_ peaks (JCPDS data card no. 41-1445).^25^ The PANI matrix, however, may be the reason for these peaks' slight displacement from their respective standard positions.^7^ Additionally, compared to the XRD of pure SnO_2_, we have observed less intense peaks and comparatively more peak broadening.

### 3.4. Surface morphological analysis

**Fig. 4:**
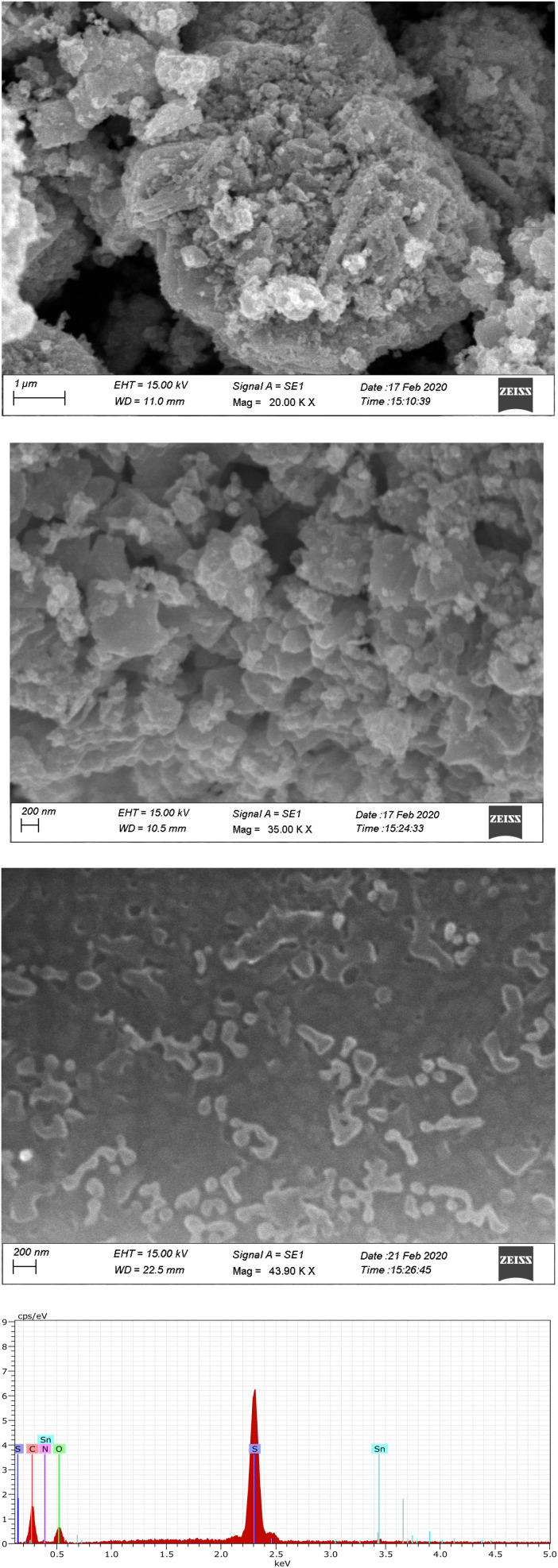
(a) SEM micrograph for tin oxide. (b) SEM micrograph for polyaniline. (c) SEM micrograph of the SnO_2_–PANI nanocomposites. (d) EDAX spectrum of the SnO_2_–PANI nanocomposites.

Fig. 4(a–c) displays the SEM micrographs of the SnO_2_–PANI nanocomposites, polyaniline, and tin oxide. The SEM image of SnO_2_ nanoparticles produced by the coprecipitation method is shown in Fig. 4(a). The particles have a spherical shape and are uniformly distributed, with sizes less than 100 nm. It is possible that the larger particles in this figure are aggregates of the smaller ones. In a study,^6^ it was found that the tin particle size decreased as pH increased from 6 to 9 but did not change after this pH value. Regarding the polyaniline particles (Fig. 4(b)), the particles grown seem to be spherical in shape, with a diameter ranging from 50 to 100 nm. The composite particles of PANI–SnO_2_ nanocomposites exhibit high dispersion and agglomeration (see Fig. 4(c)). It seems highly likely that the nanostructured SnO_2_ particles are embedded within PANI chains, based on the SEM images.^26^

The SnO_2_–PANI nanocomposites' EDX spectrum (Fig. 4(d)) shows that the only components present are tin, sulfur, nitrogen, and oxygen. There is no evidence of contamination from sodium or chlorine in the product. According to the quantitative SEM–EDX analysis, the percentage of oxygen is roughly 40.79%, whereas the percentages of tin and sulfur are roughly 20.58% and 25.63%, respectively. The observation of nearly twice as much oxygen as tin, nitrogen, and sulfur confirms the chemical composition of PANI–SnO_2_ nanocomposites.^27^

### 3.5. Thermogravimetric analysis

**Fig. 5:**
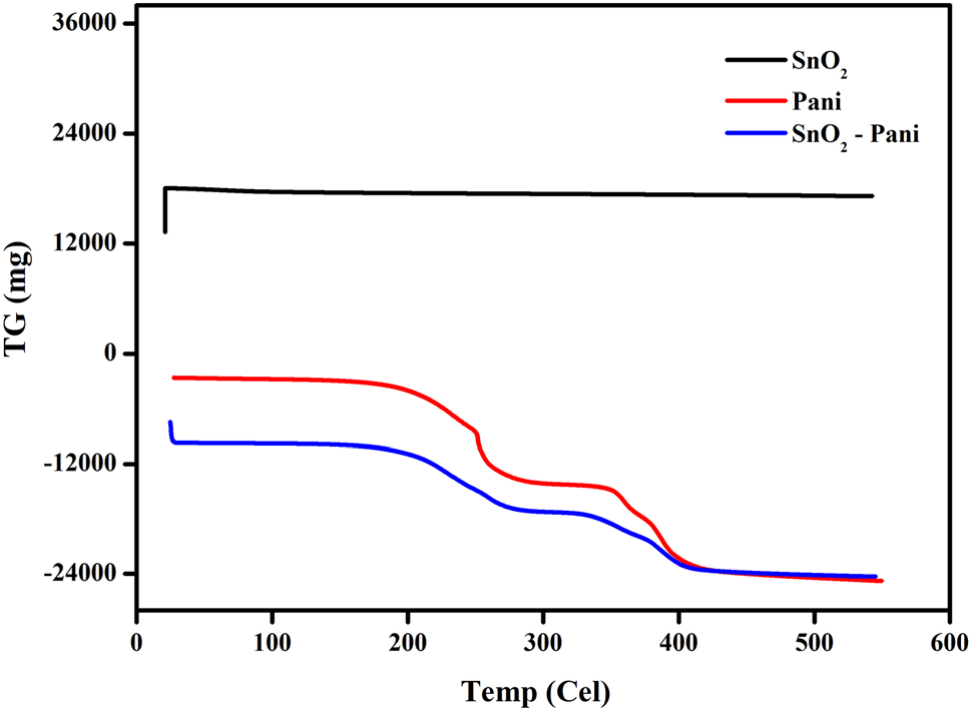
TG curves of tin oxide, polyaniline and the SnO_2_–PANI nanocomposites.

**Fig. 6:**
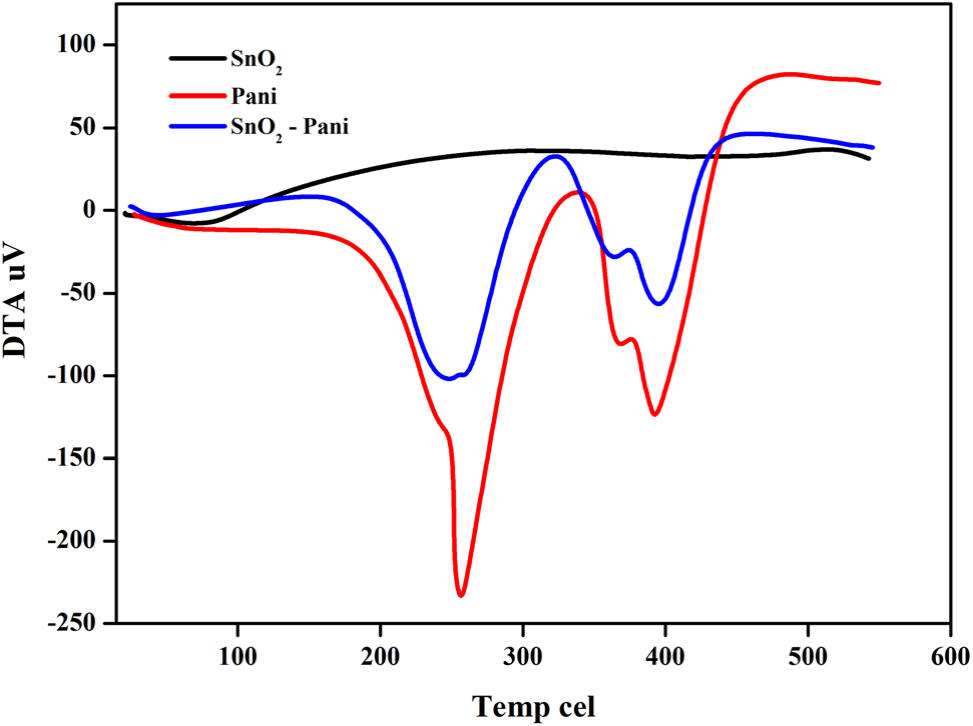
DTA curves of tin oxide, polyaniline and the SnO_2_–PANI nanocomposites.

**Fig. 7:**
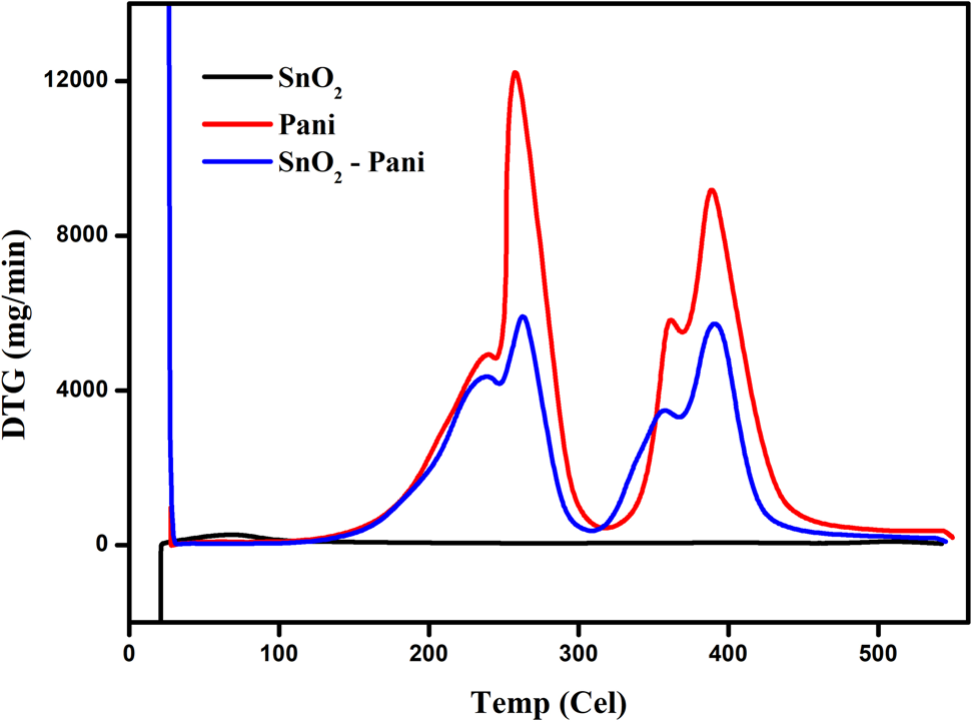
DTG curves of tin oxide, polyaniline and the SnO_2_–PANI nanocomposites.

Fig. 5–7 illustrate the TGA analysis of the dried tin oxide, polyaniline, and the SnO_2_–PANI nanocomposites at optimal concentrations. The samples were aged for 24 hours in air and then heated at a rate of 20 °C min^−1^ in a nitrogen atmosphere between 20 °C and 800 °C. In SnO_2_, the corresponding initial weight loss was 17.73 mg in the 50–80 °C temperature range. Up to 600 °C, the SnO_2_ nanoparticles remained thermally stable and did not break down. There were two phases in the weight reduction of polyaniline.^28^ The initial decrease in temperature was between 185 °C and 264 °C, which could be explained by the PANI's water evaporating. The second weight loss, which ranged from 344 °C to 406 °C, was related to ammonium persulfate breaking down. A significant weight loss of −10.11 mg for SnO_2_ modified by polyaniline was observed at temperatures between 169 °C and 268 °C. The evaporation of water from the metal oxides was responsible for this initial weight loss. The second weight loss in SnO_2_ modified with polyaniline was observed in the temperature range from 328 °C to 490 °C. This second weight loss is the result of the decomposition of ammonium per sulphate in SnO_2_ and polyaniline-modified SnO_2_ to ammonium per sulphate and polyaniline. The PANI–SnO_2_ composite decomposed slowly between 169 °C and 490 °C, while PANI broke down between 185 °C and 406 °C. This suggests that PANI had a strong interaction with the SnO_2_ surface, improving polyaniline's thermal stability.^29^

### 3.6. DLS particle size analysis

**Fig. 8:**
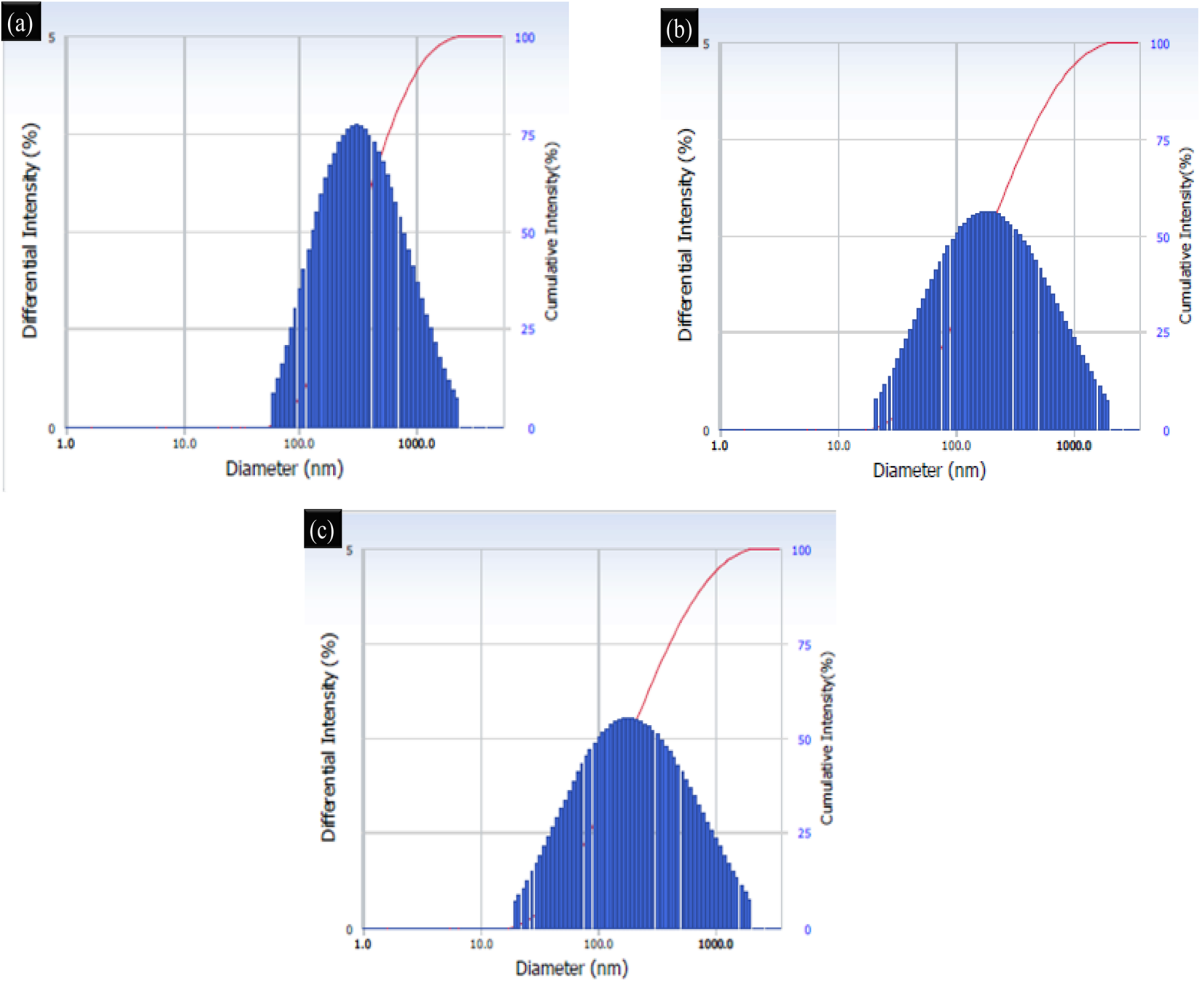
DLS particle size analysis of (a) tin oxide, (b) polyaniline and the (c) SnO_2_–PANI nanocomposites.

Dynamic light scattering (DLS) was used to measure the size distribution of the SnO_2_–PANI, polyaniline, and tin oxide nanocomposites. DLS examines how Brownian motion causes particles to move in a solution. The majority of the particle sizes in a sample are used to calculate the average size. The charge ratio and size reduction have no relationship, but the polydispersity index does describe the homogeneity of the loaded particles; the lower the index, the more uniform the particles. Using a solution of nanoparticles, the DLS method measures the intensity of scattered light.^30^ According to an analysis of the dynamic light scattering (DLS) data, tin oxide nanoparticles have an average diameter of about 100 nm in the solution with a standard composition (Fig. 8(a)). The average size distribution of polyaniline is 80–100 nm, as shown in Fig. 8(b). Similarly, Fig. 8(c) for tin oxide/polyaniline illustrates that its average size ranges between 70 and 100 nm. A proprietary high-resolution, multimodal deconvolution analysis was used for statistical analysis in order to accurately measure the diameter of the nanoparticles.

### 3.7. Atomic force microscopy

**Fig. 9:**
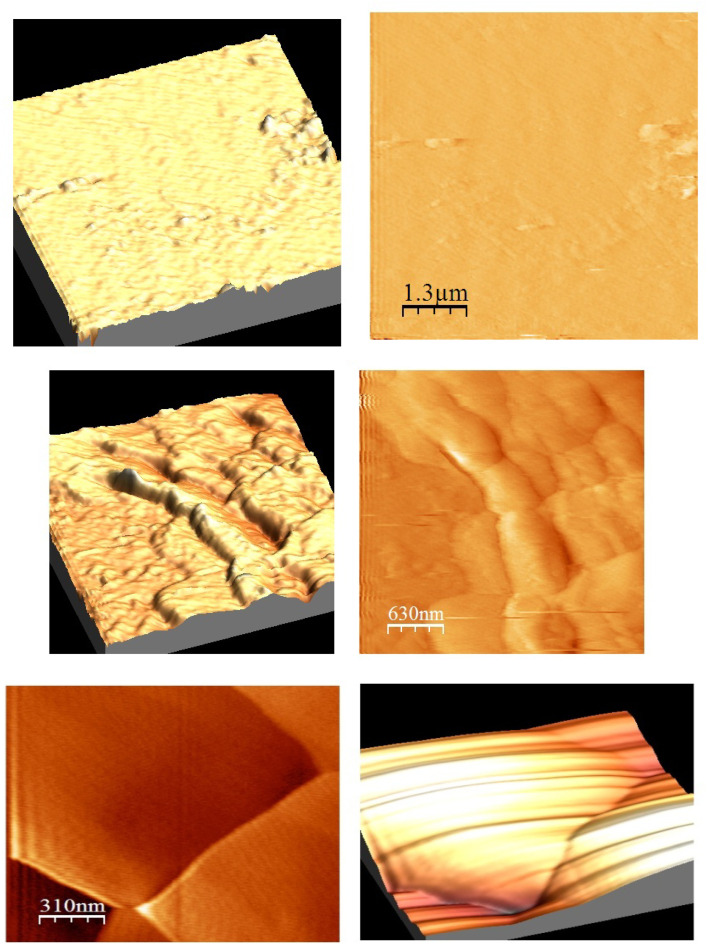
(a) AFM micrographs of the SnO_2_–PANI nanocomposites. (b) AFM micrographs of polyaniline. (c) AFM micrographs of tin oxide.

Fig. 9(a) displays the SnO_2_–PANI AFM image. The maximum grain height and maximum grain width of these nanolayers were estimated according to the exact standard AFM depth. It is evident from the image that PANI is deposited on SnO layers. Additionally, the PANI deposition has no effect on the SnO's surface area.^31^

In Fig. 9(b), the AFM image of PANI at 310 nm is displayed. The AFM image shows aggregated grains and a uniform distribution of polyaniline across the surface.^32^ A particle with distinct PANI boundaries was obtained.

Fig. 9(c) shows the AFM images of nanocrystalline films of tin oxide. The polycrystalline structure with well-shaped grains is visible in the AFM image. Due to the presence of SnO, the surface appears rough. As a result, the SnO particles' adsorption capacity is increased.

### 3.8. Gas selectivity test

**Fig. 10:**
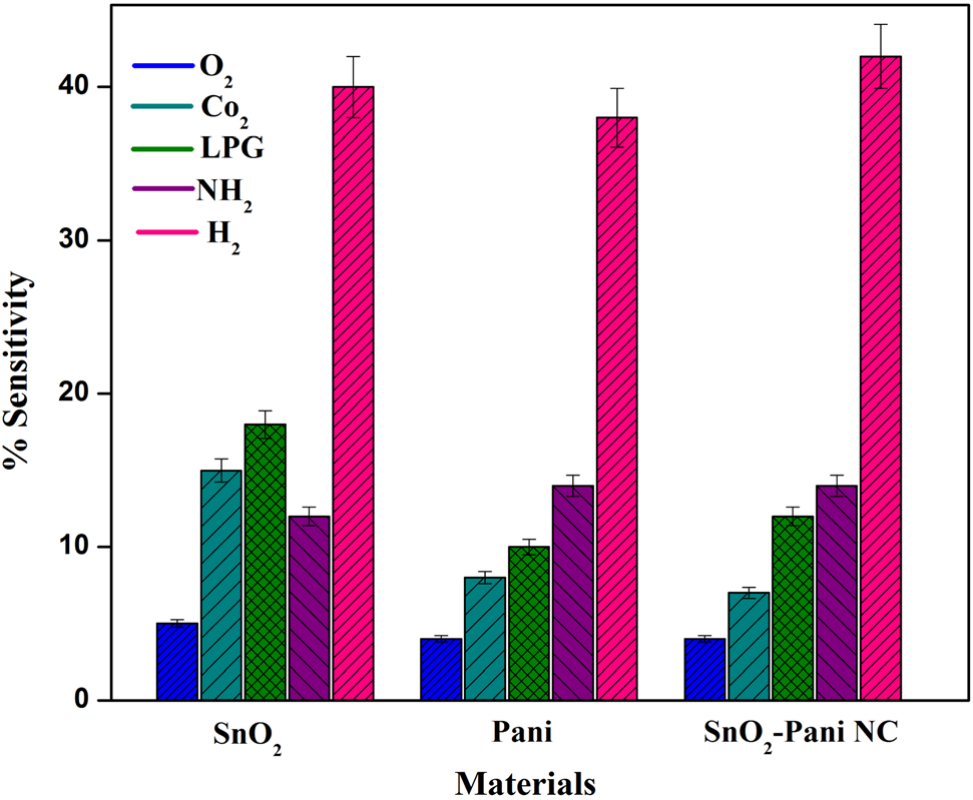
Selectivity test towards various analyte gases for SnO_2_, PANI and the SnO_2_–PANI nanocomposite.

The material used in a practical gas-sensing electrode should be able to react actively to a specific gas only. The current study assessed the materials' selectivity by introducing 50 ppm of different target gases, including NH_3_, CO_2_, H_2_, O_2_, and LPG. Fig. 10 shows a bar chart plotting the sensitivity values. For accuracy, the selectivity was measured independently three times, and the resulting values were plotted as error bars.^33^ The electrode composed of the SnO_2_–polyaniline nanocomposite exhibits a greater response to H_2_ than the other pristine and mixed composites, as can be seen in the figure. It is essential for the SnO_2_–polyaniline nanocomposite to have a high selectivity towards H_2_.

The high selectivity of the synthesized materials, including the nanocomposite (NC), toward H_2_ gas can be attributed to the strong interaction between hydrogen molecules and the surface active sites of the SnO_2_–PANI composite. Hydrogen has a smaller molecular size and higher diffusivity, which allows it to easily access and react with the adsorbed oxygen species on the sensor surface. This reaction leads to a more significant change in resistance compared to other tested gases.

Moreover, the presence of PANI in the composite enhances the surface adsorption capacity and facilitates charge transfer at the SnO_2_–PANI interface, further improving sensitivity and selectivity toward H_2_. These factors collectively contribute to the observed selective response.

#### 3.8.1. Gas sensitivity test

**Fig. 11:**
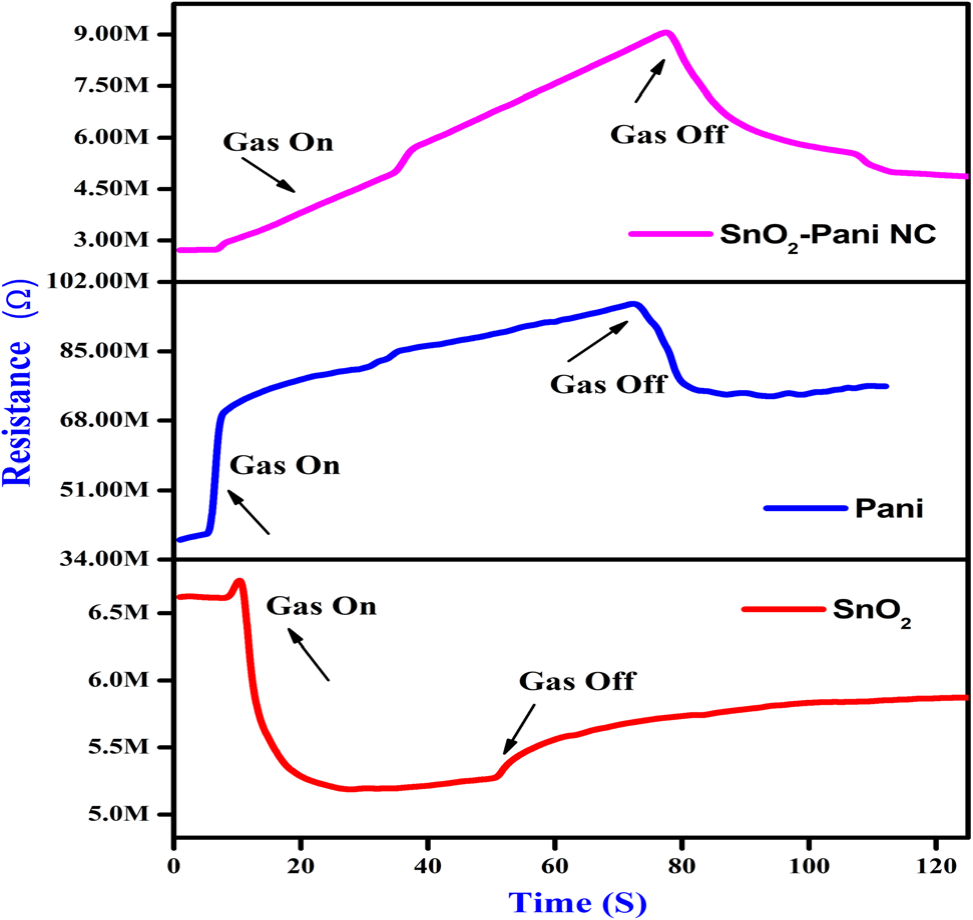
50 ppm ammonia sensing response values of the SnO_2_–polyaniline nanocomposite.

An alternative method for resolving sensitivity and selectivity problems, as well as for modern, efficient room-temperature operation, is the fabrication of metal oxide nanocomposites. The sensing parameters of the prepared materials were analysed using a Keithley 6487 picoammeter/voltage source. The following formula was used to determine the electrodes' sensing response to H_2_ and other analyte gases, such as CO_2_, LPG, and NH_2_:
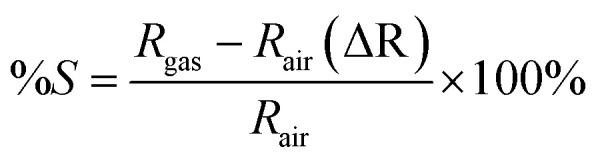


In this case, the resistances of the electrode in the presence of air and analyte gas are represented by *R*_air_ and *R*_gas_, respectively. Fig. 11 shows the response of the different prepared electrodes to 50 ppm H_2_ at 40% relative humidity. Finding the material composition that exhibits the highest sensitivity to H_2_ was the goal of the current study. An important factor in the sensing response is the atoms on the surface of the electrode material's grain boundary. Because of its high electron mobility, the SnO_2_–polyaniline nanocomposite is crucial for room-temperature sensing in this situation. One of the key parameters for assessing a material's performance for gas sensing is the sensor's response and recovery time.^34^ In order to achieve 90% of the maximum resistance change, the response and recovery time is the time required to apply input gas on and off. The response value of the SnO_2_–polyaniline nanocomposite as resistance decreased when H_2_ was present at a concentration of 50 ppm. Table 1 contains a tabulation of the observed response recovery and percentage sensitivity values. The sensing electrode was exposed to concentrations ranging from 5 to 70 ppm, and the corresponding graph is shown in Fig. 11.

**Table 1:** Comparison of the sensitivity parameters of the electrodes towards hydrogen

Materials	Response (% sensitivity)	Response time (s)	Recovery time (s)
SnO_2_	27	53	117
Polyaniline	6	91	—
SnO_2_–polyaniline nanocomposite	7	44	—

#### 3.8.2. Electrode stability test

**Fig. 12:**
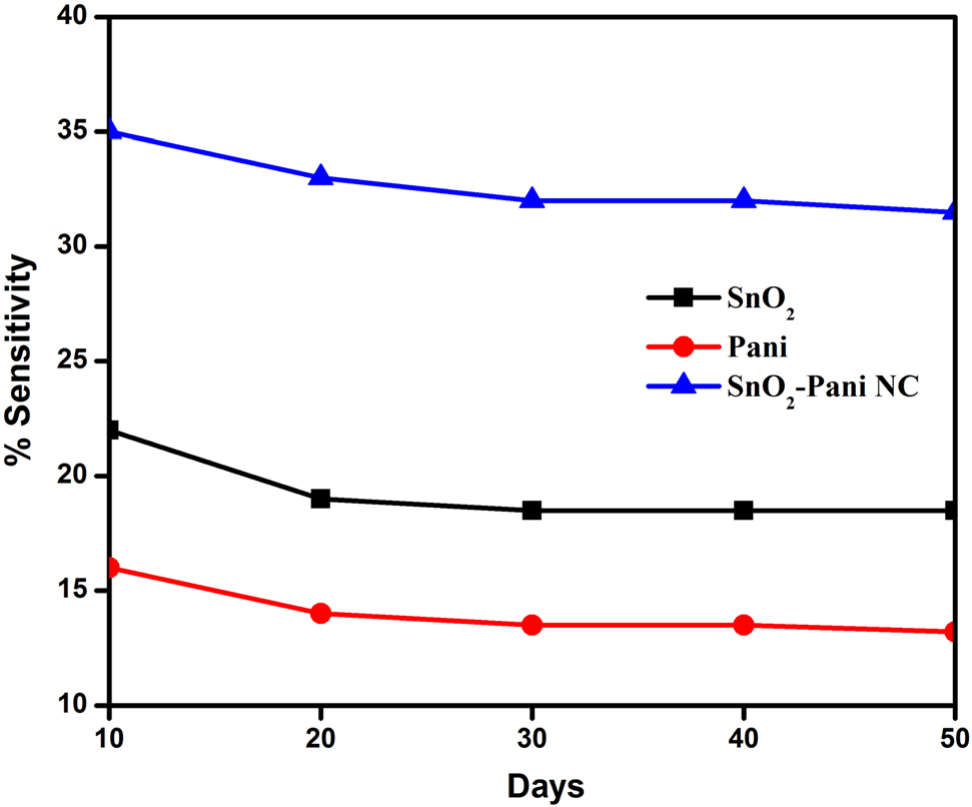
Stability of the sensing electrodes towards H_2_ for a period of 50 days.

A sensing electrode's ability to consistently reproduce the response value over an extended period of time is known as stability. Here, a 50 day test was conducted with 10 day intervals to evaluate the electrodes' stability. Fig. 12 shows the plotted responses that were recorded. SnO_2_–polyaniline nanocomposite electrodes retain 94% of the sensing values.^35^ Because the electrodes operate at room temperature, the stability test results show that the electrodes exhibit improved stability over a 50 day period. The SnO_2_–polyaniline nanocomposite exhibits the highest stability response among the sensing electrodes tested.

#### 3.8.3. Gas sensing mechanism

The sensing mechanism of metal oxide gas sensors must be understood in order to design and fabricate novel materials that perform exceptionally well during gas sensing. The gas response is primarily caused by the trapping of electrons by adsorbed molecules and band bending induced by these charged molecules, although the precise fundamental mechanisms causing this change in conductivity are still up for debate.^36^ This article provides a brief overview of the n-type metal oxide sensing mechanism in air using SnO_2_ as an example. The SnO_2_ sensing material's surface usually absorbs oxygen gas when exposed to air. From the interior of the SnO_2_ film, the adsorbed oxygen species can extract electrons. A reduced conductivity results from a depletion layer created by the negative charge trapped in these oxygen species. A reduced potential barrier height and an increased conductivity are the results of the oxygen adsorbate's ability to trap electrons, which return to the SnO_2_ film when the sensor is exposed to reducing gases. The surface contains a variety of oxygen species, ranging from atomic (O^−^ and O_2_^−^) to molecular (O_2_^−^) ions, depending on the operating temperature.^37^ In general, the molecular form predominates below 150 °C, whereas atomic species are found above this temperature.

**Fig. 13:**
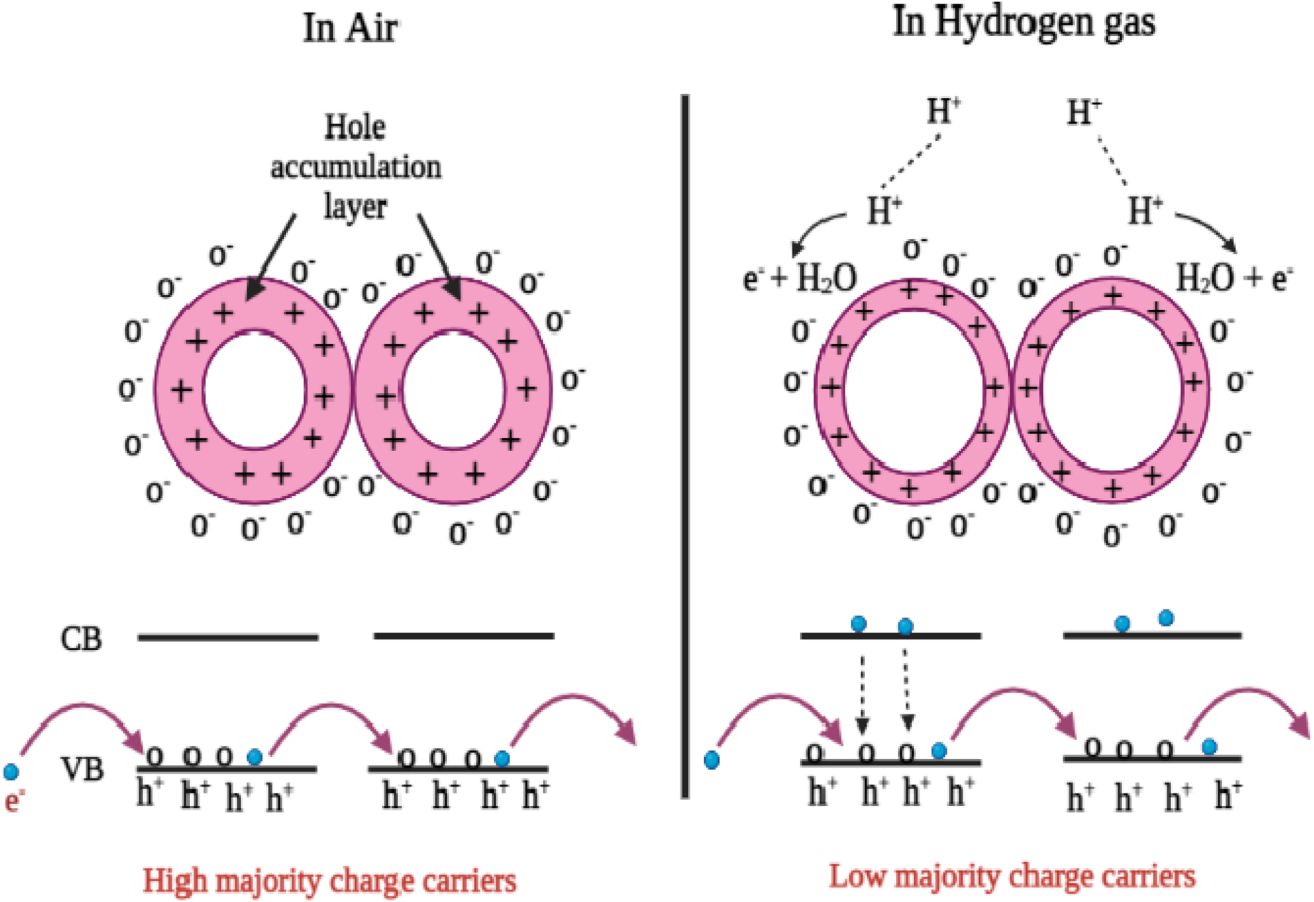
Hydrogen sensing mechanism of SnO_2_, PANI and the SnO_2_–PANI nanocomposite (a) in the presence of air and (b) in the presence of H_2_.

The surface conductivity of the metal oxides is largely determined by their total surface stoichiometry. Adsorbed oxygen ions function as surface acceptors, binding electrons and reducing surface conductivity, whereas oxygen vacancies act as donors, raising the surface conductivity. The energy diagram of different gas-phase oxygen species bound to the SnO_2_ lattice and adsorbed on its surface is depicted in Fig. 13. As the temperature rises, the reaction O_ads_^2−^ + e^−^ = 2O_ads_^−^ occurs on the SnO_2_ films. For O_ads_^−^ ions, the desorption temperature on the SnO_2_ surface is approximately 550 °C, while for O_ads_^2−^ ions, it is approximately 150 °C. Band bending and surface conductivity change in tandem with the transition, which results in an increase in surface charge density at a constant oxygen coverage.

The measurements of conductance indicate that the transition occurs gradually. Consequently, a steady and gradual change in the conductance usually occurs after a sudden change in the sensors' temperature.^38^ As the oxygen coverage reaches a new equilibrium, the adsorbed oxygen changes into a different species that can be employed in a dynamically modulated temperature measurement technique.

### 3.9. Electrochemical studies

#### 3.9.1. Cyclic voltammetry studies

**Fig. 14:**
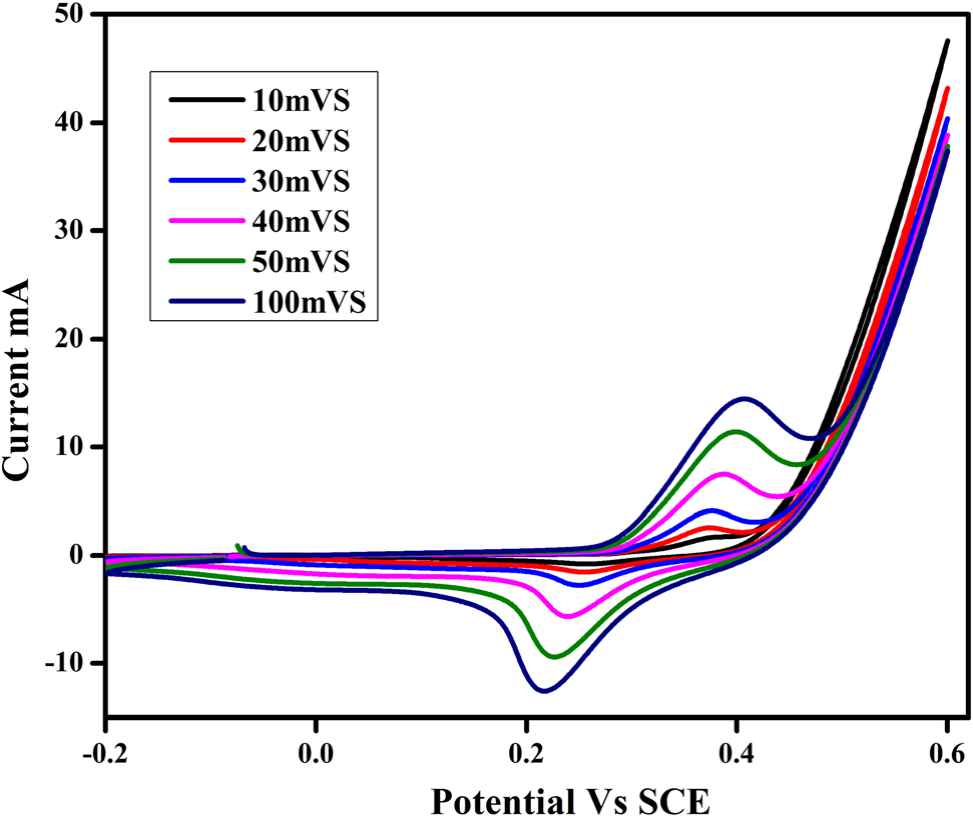
Cyclic voltammograms of the nanostructured SnO_2_–PANI nanocomposite electrode materials at different scan rates in a 3 M KOH electrolyte.

The electrochemical behavior of the produced nanocrystalline electrode materials (SnO_2_–PANI nanocomposite) for electrochemical capacitors was examined using cyclic voltammetry (CV) in a 3 M KOH solution. The CV experiments were conducted at various scan rates (10, 20, 30, 40, 50, and 100 mV s^−1^), and Fig. 14 presents the results. The potential voltage range from 0.7 to 0.4 V *vs.* SCE was used for the CV tests. The cyclic voltammograms of all the samples are oval and rectangular in shape, suggesting that, as mentioned, a typical pseudocapacitive response takes place at the graphite electrode/active material (SnO_2_–PANI nanocomposite)/electrolyte interface. These CV curves get better with increasing scan rates because they resemble a pseudocapacitive nature.^39^ Moreover, the current increases in proportion to the scan rate because at lower scan rates, the electrolyte ions have more time to enter the material's pores. At higher scan rates, however, their current dependence on the scan rate limits their collection on the electrode's outermost surface. Eqn (2) was utilized to calculate the values of specific capacitance.

Table 2 displays the specific capacitance values computed for the nanostructured SnO_2_–PANI nanocomposite material at various scan rates. At a scan rate of 10 mV s^−1^, the SnO_2_–PANI nanocomposite demonstrates the highest specific capacitance (150.6 F g^−1^) among the electrode materials.

**Table 2:** Specific capacitance data obtained for the nanostructured SnO_2_–PANI nanocomposite electrode materials

Specific capacitance values at different scan rates						
Electrode material	100 mV s^−1^	50 mV s^−1^	40 mV s^−1^	30 mV s^−1^	20 mV s^−1^	10 mV s^−1^
SnO_2_-PANI nanocomposite	100.53 F g^−1^	120.4 F g^−1^	140.6 F g^−1^	125.3 F g^−1^	134.94 F g^−1^	150.6 F g^−1^

#### 3.9.2. Galvanostatic charge–discharge studies

**Fig. 15:**
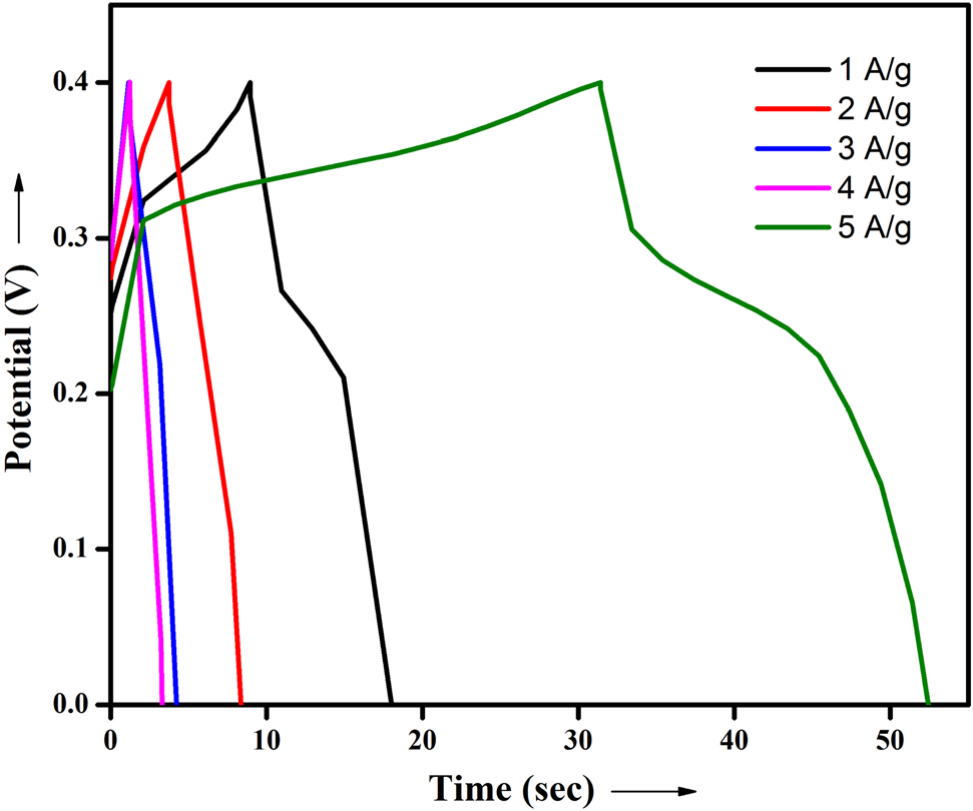
Galvanostatic charge–discharge curves obtained for the nanostructured SnO_2_–PANI nanocomposite electrode materials at different current densities.

The electrochemical properties of the electrode materials, like SnO_2_–PANI nanocomposite, were investigated through galvanostatic charge–discharge (GCD) tests conducted in a 3 M KOH solution. The outcomes are shown in Fig. 15. At current densities ranging from 1 to 5 Ag^−1^, the charge–discharge curves of the electrode materials based on the SnO_2_-PANI nanocomposite were measured. Every GCD curve displayed a symmetrical triangle, suggesting that all samples exhibited an optimal capacitor behavior.^40^

#### 3.9.3. Impedance spectroscopic studies

The charge transport kinetics of the electrode materials were investigated using EIS measurements. According to reports, the Nyquist plot provides information on interfacial effects, electrode–electrolyte interactions, and equivalent series resistance (ESR). An EIS analysis of the SnO_2_–PANI nanocomposite electrode materials was performed in the frequency range of 1 Hz to 1 kHz using an amplitude of V.

**Fig. 16:**
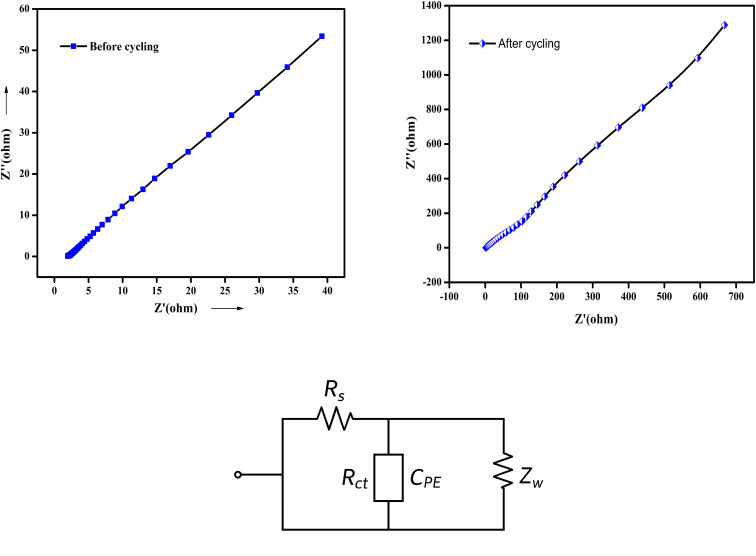
Nyquist plots obtained for the nanostructured SnO_2_–PANI nanocomposite electrode materials and the corresponding equivalent circuit diagram.

The Nyquist plots generated for the SnO_2_–PANI nanocomposite nanostructured electrode materials are displayed in Fig. 16. All Nyquist plots display a line that is almost exactly parallel to the fictitious components.^41^ The described straight line obtained in the low-frequency range indicates that all four samples exhibit a perfect capacitance behavior. Moreover, a combination of electrode/electrolyte resistance, the active material's internal resistance, electrolyte interfacial resistance, and the electrode/current collector's contact resistance makes up the EIS produced by the intercept at the actual impedance axis. The straight line slope in the low-frequency area of the Nyquist plot corresponds to the Warburg behavior caused by rapid ion diffusion across the electrolyte/electrode interface. Consequently, we can say that supercapacitors can exhibit ideal blocking behavior at high frequencies and capacitive behavior at low frequencies.^42^ This research may provide a well-defined process for fabricating electrode materials for high-performance supercapacitors based on SnO_2_–PANI nanocomposite.

The EIS spectra of SnO_2_, PANI, and the SnO_2_–PANI nanocomposite are shown in Fig. 16, with the corresponding equivalent circuit provided in the inset. The semicircle observed at high frequencies represents the charge transfer resistance (*R*_ct_), while the linear portion at low frequencies corresponds to Warburg diffusion. The SnO_2_–PANI composite exhibits a smaller *R*_ct_ than pure SnO_2_, indicating enhanced electron transfer due to the conductive PANI network. The slope of the low-frequency region suggests improved ionic diffusion, consistent with the porous morphology observed in SEM images. These results confirm that the composite structure facilitates both electronic and ionic transport, contributing to improved electrochemical performance.

### 3.10. Photocatalytic activity

**Fig. 17:**
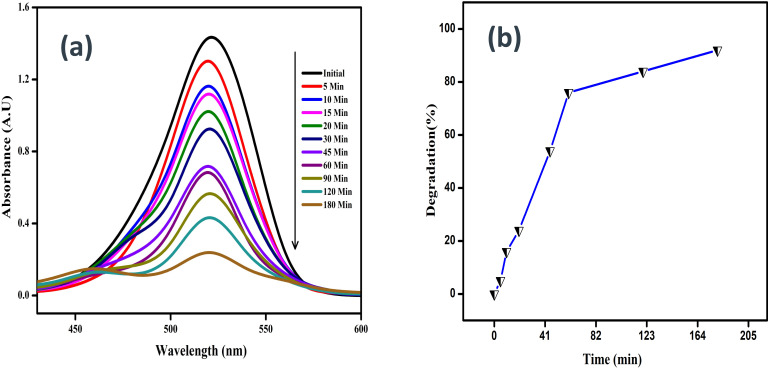
Photocatalytic degradation of rhodamine dye using the SnO_2_–PANI nanocomposite: (a) UV-visible spectra and (b) percentage degradation.

Fig. 17 shows the photocatalytic degradation of the dye molecule rhodamine through chemically assisted synthesis. An SnO_2_–PANI nanocomposite is generated at room temperature when it is exposed to 540 nm UV radiation. The lowest peak, which was detected at 540 nm, represents the dye molecule's breakdown during UV irradiation, as evidenced by the Rhodamine dye molecule's decreasing level with prolonged UV exposure hours.^43^

This indicates that the semiconductor is excited by UV light to produce free radicals during the dye degradation process. The excited electrons subsequently move from the valence band to the conduction band, generating high-energy electron–hole pairs that transfer to adsorbed species on the semiconductor, resulting in heterogeneous photocatalysis. This process accounts for 92% of the dye molecule's degradation rate using the chemically created SnO_2_–PANI nanocomposite within 180 minutes.^44^ After the photocatalytic activity is finished in three hours, the rhodamine dye (0.2 L) is successfully destroyed. It is worth mentioning that the SnO_2_–PANI nanocomposite exhibits good performance as an adaptable photocatalytic nanoparticle.

#### 3.10.1. Photocatalytic charge separation mechanism

In SnO_2_–PANI nanocomposites, the enhanced photocatalytic activity is primarily attributed to the effective separation of photo-generated electron–hole pairs, facilitated by the synergistic interaction between SnO_2_ and PANI:

(1) Light absorption and excitation

Upon illumination, SnO_2_ absorbs photons with an energy greater than its band gap (≈3.6 eV) to generate electron–hole pairs:SnO_2_ + *hν* → e_CB_^−^ + *h*_VB_^+^

(2) Charge transfer at the SnO_2_–PANI interface

Polyaniline (PANI), as a conductive polymer with delocalized π electrons, acts as an electron acceptor or transporter, facilitating the transfer of photo-generated electrons from SnO_2_ to PANI.

This transfer suppresses electron–hole recombination, prolonging the lifetime of charge carriers.

(3) Formation of reactive oxygen species (ROS)

The transferred electrons on PANI reduce dissolved O_2_ to superoxide radicals (O_2_˙^−^), while holes in SnO_2_ oxidize water or OH^−^ to hydroxyl radicals (OH˙):e^−^ + O_2_ → O_2_˙^−^h^+^ + H_2_O/OH^−^ → OH˙

These ROS are responsible for the photocatalytic degradation of organic pollutants or microbial inactivation.

In summary, the SnO_2_–PANI nanocomposite exhibits improved photocatalytic performance due to rapid electron transfer from SnO_2_ to PANI, reduced recombination of electron–hole pairs, and increased generation of reactive oxygen species at the interface.

### 3.11. Toxicity study of the photocatalyst SnO_2_–PANI nanocomposite

**Fig. 18:**
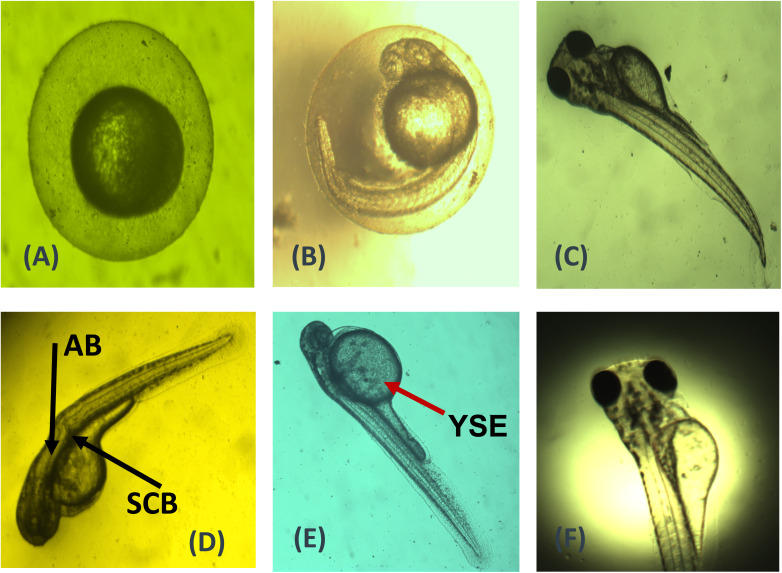
Imaging of SnO_2_–PANI nanocomposite in zebrafish embryos and larvae. (A–D) denotes the control and mortality images of the SnO_2_–PANI nanocomposite, (E and F) illustrate Yolk Salk Edema (YSE) at 48 hours, and (D) shows Spinal Card Bend (SCB) and Axis Bent (AB) at 72 hours.

**Fig. 19:**
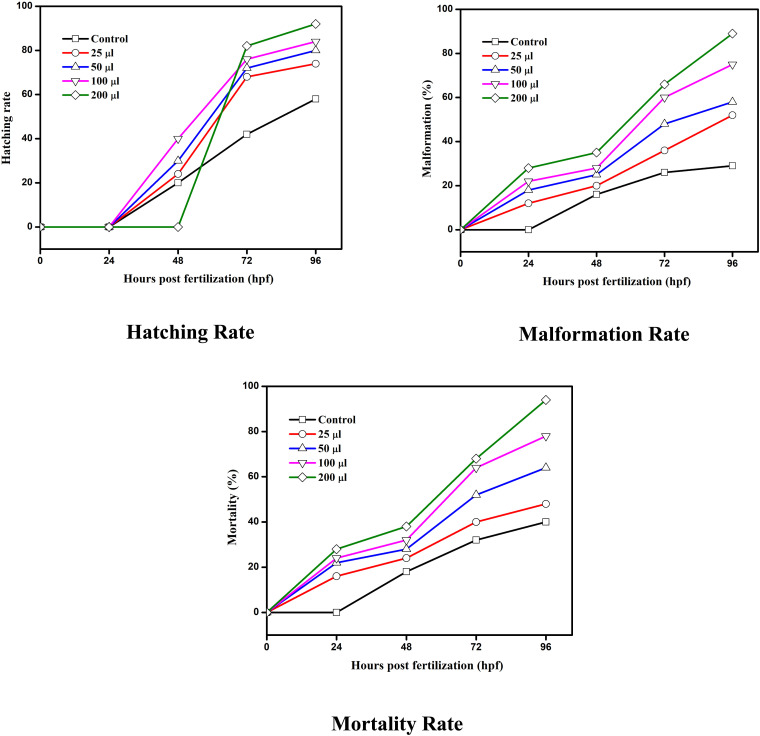
Effects of the SnO_2_–PANI nanocomposite on the hatching rate, malformation rate and mortality rate of zebrafish embryos from 24–96 hpf.

The SnO_2_–PANI nanocomposite exhibited notable toxicity toward the eggs of adult zebrafish, as shown in Fig. 18 and 19. During 48 hours of exposure, the eggs of adult zebrafish showed high hatching rates at concentrations of 100 and 200 µL, corresponding to the LC50 value. When the concentration of the SnO_2_–PANI nanocomposite is increased, the degree of deformity also increases daily.^45^ After 96 hours, the eggs' deformity reaches a high point at a concentration level of 200 µL. The SnO_2_–PANI nanocomposite exhibits similar death rates, indicating that at a concentration of 200 µL, significant mortality occurs at 96 hours, while eggs subjected to the control level also experienced mortality at 48 hours.^46^ These variations make it very clear that the toxicity of the nanocomposite depends on the particle's size, shape, capping agent, and stability and the aquatic medium's quality, in addition to the amount applied.

### 3.12. Antibacterial activity

**Fig. 20:**
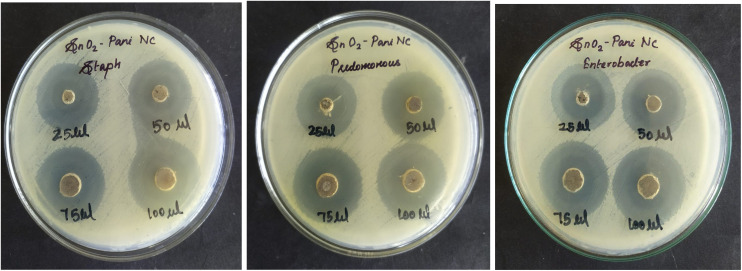
Zone of inhibition of the SnO_2_–PANI nanocomposite using various bacterial strains.

When the synthesized SnO_2_–PANI nanocomposite was tested against the bacterial strains *Pseudomonas*, *Enterobacter*, and *Staphylococcus aureus*, the inhibition zone values were obtained. At various concentrations (10–100 µL), the antibacterial activity of SnO_2_–PANI nanocomposite was evaluated; 25 µL of the nanocomposite showed antibacterial activity at all concentrations.^47^ There was no antibacterial effect in the concentration range of 10–20 µL. The minimum inhibitory concentration (MIC) was therefore determined to be 25 µL. Table 3 and Fig. 20 present the images and average values of the inhibition zones, respectively.

**Table 3:** Zone of inhibition of the SnO_2_–PANI nanocomposite against selected bacterial strains

Concentration	Zone of inhibition (diameter in cm)		
	*Enterobacter*	*Staphylococcus aureus*	*Pseudomonas*
	SnO_2_–PANI nanocomposite	SnO_2_–PANI nanocomposite	SnO_2_–PANI nanocomposite
25 µL	1.6	1.4	1.0
50 µL	1.8	1.6	1.2
75 µL	2.2	2.0	1.4
100 µL	2.4	2.5	1.8

The table displays the zone of inhibition for the SnO_2_–PANI nanocomposite (C). The antibacterial activity of the SnO_2_–PANI nanocomposite suspension against both Gram-positive and Gram-negative bacteria was found to be high and comparable, as indicated by Table 3. Due to its size, the SnO_2_–PANI nanocomposite can readily penetrate bacterial nuclei and exhibit a sizable and remarkable surface area, where the greatest amount of contact with bacteria occurs.^48^ This may be the cause of the best antibacterial activity of these SnO_2_–PANI nanocomposite materials (Table 4).

**Table 4:** Comparison table of contents

Study	Synthesis method	Application and key performance/observation	Remarks/limitations	References
SnO_2_–PANI/Pd nanocomposite (nanosheets + PANI doped with Pd)	Hydrothermal SnO_2_ → combine with PANI + Pd (likely *in situ* polymerization)	Hydrogen (H_2_) sensing at room temperature (50–400 ppm)—enhanced sensitivity *vs.* individual components	Good proof-of-concept but uses Pd (noble metal), which increases the cost. No long-term stability/cycle tests reported	6
Ag–SnO_2_/PANI composite nanofibers	Electrospinning + calcination for Ag–SnO_2_; dip-coating PANI by *in situ* polymerization	Hydrogen sensing—sensitive at ≈42 °C (much lower than bare SnO_2_)	Demonstrates low-temperature operation but requires Ag doping. No report on selectivity, cross-gas interference, and long-term cycling	49
SnO_2_/PANI electrospun nanofibers (no catalytic dopants)	Electrospinning composite fibers; characterized by XRD, SEM/EDX, UV-vis analyses; tested for H_2_ and CO sensing at ∼35 °C; fast response/recovery (<30 s)	Gas sensing (H_2_, CO) and promising sensitivity and response kinetics at low temperature	Encouraging for low-cost sensors; but sensitivity *vs.* concentration curve, long-term stability, reproducibility over many cycles not fully explored	7
SnO_2_/PANI nanoparticles (hydrothermal + PANI adsorption) for Cr(vi) reduction under visible light	Hydrothermal SnO_2_; PANI adsorption in DMF; composite dried and characterized (XRD, XPS, TEM, UV-vis DRS, EIS analyses)	Photocatalytic reduction of Cr(vi) under visible light—up to 23× rate compared to bare SnO_2_	Demonstrates visible-light-driven photocatalysis *via* p–n heterojunction; but only Cr(vi) reduction tested; limited data on reusability, stability, and extension to other pollutants/dyes	50
SnO_2_/PANI nanocomposites (general)—review of photocatalytic behavior	Analysis of prior works showing that coupling PANI (p-type) with SnO_2_ (n-type) improves visible-light absorption and charge separation, enhancing photocatalytic activity compared to SnO_2_ alone	Photocatalysis (dyes and pollutants)—better performance than bare SnO_2_; more environmentally friendly byproduct profile	Encouraging rationale; but detailed experimental parameters vary widely; systematic comparison lacking	51
SnO_2_-based (not necessarily PANI) composites for antibacterial and multifunctional uses	Many studies show SnO_2_ NPs or doped SnO_2_ have antibacterial activity (gram +/−) *via* ROS generation, often light-activated	Antibacterial/antimicrobial potential; sometimes combined with photocatalysis or energy storage (in other composites)	For SnO_2_–PANI specifically, antibacterial action remains almost unexplored. Recent (2025) work reports synthesis but does no biological results reported	52

## 4. Conclusion

In this research, binary nanocomposites (SnO_2_–PANI NCs) were prepared using a polymer (polyaniline) and SnO. The synthesized nanocomposites were characterized using various techniques like UV-vis spectroscopy, X-ray diffraction, Fourier transform infrared spectroscopy, particle size analysis, scanning electron microscopy, elemental analysis, thermogravimetric analysis and atomic force microscopy. The nanocomposites underwent various studies like gas sensing, electrochemical, supercapacitor, photocatalytic, antibacterial and toxicity studies.

The UV band gap study revealed that the band gap energy of PANI–SnO nanoparticles was about 2.3 eV. The FTIR study showed that the functional moieties that were involved in the nanoparticle synthesis and the presence of nanoparticle peaks in the solution. The particle size analysis showed that the particles' sizes were in the range of 70–110 nm. The SEM–EDAX analysis depicted that the particles were mostly spherical and agglomerated, and the EDAX showed the presence of Sn in the nanoparticle solution with a strong band at 3.5 keV. The nanoparticles showed good sensitivity, detecting CO_2_ at 50 ppm. The electrode material was highly sensitive to H_2_ compared to other gases like O_2_, CO_2_, NH_3_, and LPG. The electrode material was stable for 50 days with a 94% sensing capacity. The electrochemical study showed that the particles had a high current density and specific surface area. The bimetallic nanoparticle was used to degrade the rhodamine dye in a photocatalytic chamber. The nanoparticles removed 92% of the dye in 180 min with a high removal efficiency. The toxicity test was performed to test the toxic nature of the photocatalyst, and the nanoparticles showed mild toxicity to the model animal. The antibacterial test was conducted for Gram-positive and Gram-negative bacteria, and the particles showed good activity compared to the control.

## Author contributions

Dr E. Amutha – writing and review, manuscript preparation and revision, methodology, validation, conceptualization, investigation; Dr S. Rajaduraipandian – implementation of experiments, data collection, sample analysis, writing and review; T. Madhumitha – software, data collection; G. Annadurai – supervision, investigation, implementation of experiments and manuscript revision; Vijayalakshmi Shankar – implementation of experiments, validation and review.

## Conflicts of interest

The authors declare that there is no conflict of interest.

## Data Availability

The datasets used and/or analyzed during the current study are available from the corresponding author upon reasonable request.
